# Local Augmented Angiotensinogen Secreted from Apoptotic Vascular Endothelial Cells Is a Vital Mediator of Vascular Remodelling

**DOI:** 10.1371/journal.pone.0132583

**Published:** 2015-07-06

**Authors:** Shyh-Jong Wu, Mathilde Soulez, Ya-Hui Yang, Chih-Sheng Chu, Shih-Chuan Shih, Marie-Josée Hébert, Mei-Chuan Kuo, Ya-Ju Hsieh

**Affiliations:** 1 Department of Medical Laboratory Science and Biotechnology, Kaohsiung Medical University, Kaohsiung, Taiwan; 2 Institute for Research in Immunology and Cancer, Université de Montréal, Montréal, QC, Canada; 3 Department of Occupational Safety and Hygiene, Fooyin University, Kaohsiung, Taiwan; 4 Division of Cardiology, Department of Internal Medicine, Kaohsiung Medical University Hospital, Kaohsiung, Taiwan; 5 Department of Internal Medicine, Kaohsiung Municipal Ta-Tung Hospital, Kaohsiung, Taiwan; 6 Graduate Institute of Medicine, Kaohsiung Medical University, Kaohsiung, Taiwan; 7 Research Centre, Centre Hospitalier de l'Université de Montréal (CRCHUM), Hôpital Notre Dame and Université de Montréal, Montréal, QC, Canada; 8 Department of Neurology, Kaohsiung Medical University Hospital, Kaohsiung, Taiwan; 9 Department of Medical Imaging and Radiological Sciences, Kaohsiung Medical University, Kaohsiung, Taiwan; University of Sassari, ITALY

## Abstract

Vascular remodelling is a critical vasculopathy found in atheromatous diseases and allograft failures. The local renin angiotensin system (RAS) has been implicated in vascular remodelling. However, the mechanisms by which the augmented local RAS is associated with the initial event of endothelial cell apoptosis in injured vasculature remain undefined. We induced the apoptosis of human umbilical vein endothelial cells (HUVECs) and vascular smooth muscle cells (VSMCs) through serum starvation (SS). After the cells were subjected to SS, we found that the mRNA expression of angiotensinogen (AGT) was increased by >3-fold in HUVECs and by approximately 2.5-fold in VSMCs. In addition, the expression of angiotensin-converting enzyme (ACE) mRNA was increased in VSMCs but decreased to 50% in HUVECs during the same apoptotic process. Increases in the expression of AGT protein and angiotensin II (Ang II) were found in a serum-free medium conditioned by HUVECs (SSC). The increased Ang II was suppressed using lisinopril (an ACE inhibitor) treatment. Moreover, the activation of ERK1/2 induced by the SSC in VSMCs was also suppressed by losartan. In conclusion, we first demonstrated that the augmented AGT released from apoptotic endothelial cells acts as a vital progenitor of Ang II to accelerate vascular remodelling, and we suggest that blocking local augmented Ang II might be an effective strategy for restraining intimal hyperplasia.

## Introduction

Vascular remodelling (or neointima formation) is a common feature of vasoocclusive disorders (e.g., atherosclerosis) [1], restenosis after balloon angioplasty [2], and percutaneous transluminal coronary angioplasty [3] as well as the main cause of allograft failure in transplant organs (e.g., kidney transplantation) [4]. Although it is characterised by vascular smooth muscle cell proliferation and extracellular matrix deposition in the vascular intimal layer [5], the molecular mechanisms leading to vascular remodelling in vasculopathy have yet to be clearly delineated.

The renin angiotensin system (RAS) is an integrated system of peptides (angiotensinogen [AGT], the precursor of angiotensin II [Ang II]), enzymes (angiotensin-converting enzyme 1 [ACE1], ACE2, and renin), and membrane receptors (Ang II receptors, AT1R, and AT2R). This system mainly regulates the blood pressure and maintains hydromineral homeostasis. Several RAS components have been identified as participating in the process of vascular remodelling after vascular endothelial injury [6]. At increased levels, Ang II, the major bioactive product of the RAS, elicits several deleterious effects on vessel walls and has been implicated in the pathogenesis of atheromatous diseases [7]. Increased mRNA expression of the local RAS has been detected in the neointima of the injured vasculature [8], and the inhibition of RAS activation could alleviate stress-induced endothelial dysfunction and increase neointima formation after vascular injury [9, 10]. These observations suggest that continually increased local RAS production contributes to the pathophysiological process of vascular remodelling. However, few studies have clarified the exact source of local augmented Ang II.

Because endothelial cells (ECs) function on the surface of the vasculature and encounter continual dynamic challenges from the blood stream in a vascular vessel, the apoptosis of ECs induced by continued stresses (e.g., chronic inflammation stimulated by nonimmune or immune insults) [11, 12] has been identified as an initial event in the development of vascular remodelling in atheromatous diseases [13, 14]. For instance, our recent report showed that the perlecan fragment LG3 released from apoptotic ECs could induce the migration and proliferation of vascular smooth muscle cells (VSMCs) in the intima layer and aggregated migrated tissue in the inner lining of the vessel [14]. Nevertheless, because of the extreme difficulty of detecting the differential expression of AGT and ACE protein in the thin lining of vascular ECs in the apoptotic state in vivo, it is difficult to determine whether continual local augmented Ang II production inducing vascular remodelling is associated with the apoptosis of ECs and VSMCs. Thus, on the basis of our previous findings [14, 15], this study further investigated the mechanisms of paracrine AGT and ACE when ECs and VSMCs are under apoptotic stress in vitro.

## Materials and Methods

### Ethics statement

All animal experiments were approved by the Comité Institutionnel de Protection des Animaux (CIPA) of the Centre Hospitalier de l'Université de Montréal (CHUM). Mice were fed standard chow diet and housed in temperature- and humidity controlled room (22±3°C and 60±5% respectively) that a 12:12-hr light-dark cycle was maintained. All surgical procedures were performed under anesthesia with an intramuscular injection of 100 μl of a ketamine mixture (10 μl ketamine HCL, 7.6 μl xylazine, 2.4 μl acepromazine maleate, and 10 μl PBS). Euthanasia was achieved by 100% carbon dioxide inhalation, followed by cervical dislocation.

### Cell culture

Human umbilical vein endothelial cells (HUVECs) and rat aortic smooth muscle cell line A7r5 (denoted as VSMC) were purchased from BCRC (Taiwan) and ATCC (USA), and were cultivated as we described previously [15]. Briefly, HUVECs were cultivated on flasks or petri dishes coated with 0.2% gelatin in medium M199 supplemented with 10% fetal bovine serum (Gibco BRL, Gaithersburg, MD, USA), 15 mg/L ECGS (Upstate, NY, USA) in an atmosphere of 5% CO_2_ at 37°C. The medium was changed every 2–3 days until the cells reached confluence. The experiments on HUVECs were conducted between cell passages 3 and 5. Those passages of cells were grown in 6-well plates coated with gelatin, and the cells were used for treatments. A7r5 cells were cultured in Dulbecco modified Eagle medium supplemented with 10% fetal bovine serum, penicillin, and streptomycin at 37°C in 5% CO_2_, and passages 3 to 12 were used in the experiments.

### Generation of conditioned media and ELISA assay

The serum-free medium preconditioned using ECs (SSC) was collected by starving HUVECs with serum free medium for 4 h as we described previously [15]. After centrifugation (4000 xg for 15 minutes at 4°C), all supernatants were preserved at -20°C before they were used for the assay (ex. ELISA assay or treating A7r5 cells). The ACEi-SSC was the serum-free medium preconditioned using ECs with a parallel treatment of 100 μM ACE inhibitors (lisinopril or benazepril, purchased from Sigama) for 4 h, and was harvested as serum starvation (SS) +Lis and SS+Ben. The concentration of Ang II in SS, SSC, and ACEi-SSC was detected using the commercial Ang II ELISA kit (CUSABIO BIOTECH Co., Ltd., Wuhan, China).

### Cell viability assay

PrestoBlue Cell Viability Reagent (Invitrogen), a ready-to-use cell-permeable resazurin-based solution, was applied to examine the cell viability in serum-starvation experiments by using the reducing power of living cells to quantitatively measure the proliferation of cells. When added to cells, the PrestoBlue reagent is modified by the reducing environment of the viable cell and turns red and highly fluorescent. The colour change can be detected using fluorescence or absorbance measurements. Briefly, a 96-well plate containing the cells and the compounds to be tested was prepared. The PrestoBlue reagent was added directly to the cells and incubated at 37°C for ≥10 minutes. The plate was then transferred to a fluorescence reader to measure the signal. All experiments were performed in sextuplicate. Results were evaluated by plotting the signal versus the compound concentration.

### Animals and aorta transplantation procedures and immunohistochemistry

Orthotopic transplantation of aortic grafts was performed between fully MHC-mismatched BALB/c donors and C57Bl/6 recipients in absence of immunosuppression as we described previously [14]. Paraffin-embedded sections were stained with antibodies against activated caspase-3 (Cell Signaling Technology, Beverly, MA, USA); immunohistochemical staining was performed using the standard avidin-biotin-peroxidase complex method (ABC Staining System, Santa Cruz Biotechnology, Inc.).

### Real-time qPCR assays for gene expression

Total RNA was used in real-time qPCR to quantify the amount of AGT and ACE mRNA expressed in HUVECs and VSMCs according to the regular protocol described briefly as follows. Total RNA was isolated from cultured cells exposed to the various experimental conditions with Trizol reagent (Invitrogen) for 6, 12, or 24 h. An equal amount of RNA was used as a template in reverse transcription reaction performed with oligo (dT) primers and MMLV (Invitrogen) to obtain cDNA. PCR reactions were performed using the ABI 7500 real-time PCR system (Invitrogen). The mRNA of GAPDH was used as the control for sample loading and mRNA integrity. The sense and antisense primers for human AGT mRNA quantification are 5’-TCCAGCCTCACTATGCCT-3’ and 5’-GCGGTCATTGCTCAATTTTT-3’; for human ACE mRNA are 5’-CGCTGAAACCGCT GTA-3’ and 5’-TGGGGGAGTTGTACCAGGAG-3’; for human GAPDH mRNA are 5’-GAG TCAACGGATTTGGTCGT-3’ and 5’-TTGATTTTGGAGGGATCTCG-3’; for rat AGT mRNA are 5’-CCT CGC TCT CTG GAC TTA TC-3’ and 5’-CAG ACA CTG AGG TGC TGT TG-3’; for rat ACE mRNA are 5’-TCCTATTCCCGCTCATCT-3’ and 5’-CCAGCC CTTCTGTACCATT-3’; and for rat GAPDH mRNA are 5’-GCAAGTTCAACGGCACAGTC AAG-3’ and 5’-ACATACTCAGCACCAGCATCACC-3’.

### Plasmid constructs and transient transfection and luciference assay

The reporter plasmid pAGTpro-SEAP was constructed as previously described [16]. In addition, the promoter fragment of human ACE (approximately -760 to +130) was amplified through PCR and cloned into a pSEAP-Bas vector to obtain pACEpro-SEAP, as described in our previous report [17]. The strain with a forward direction of ACE promoter pACEpro-SEAP was obtained. VSMCs were freshly subcultured at a density of 2 × 10^5^ cells on 6-well cassettes and transfected using a transient liposome cotransfection protocol (TransFast Transfection Reagent, Promega, Charbonnières-les-Bains, France) with 2.4 μg of a reporter plasmid and 0.6 μg of pGL4 Luciferase Reporter Vector, which is for firefly luciferase gene expression as an internal control. All samples were run in quadruplicate in at least 3 different experiments. The conditioned cell culture medium was collected for measuring the SEAP activity of each construct by using a Great EscAPe SEAP Chemiluminescence Detection kit (BD Biosciences Clontech) and Luminometer (Labsystems Luminoskan). The luciferase assay of pGL4 firefly luciferase in cell lysate was performed according to the manufacturer’s protocol for the commercial kit (Promega).

### Immunoblotting

Immunoblotting analysis was used to detect the phosphor-ERK 1/2, total ERK 1/2, beta-actin, and AGT expression. In brief, the cells (1 × 10^6^ cells/4 mL in a 6-well plate) treated with the various experimental medium for the setting period were collected and washed with PBS. Subsequently, cellular proteins were prepared and separated on 10% SDS-polyacrylamide gel electrophoresis (SDS-PAGE) gels and blotted on a pure nitrocellulose blotting membrane (Pall, USA). The membrane was incubated in a fresh blocking buffer (0.1% Tween 20 in Tris-buffered saline, pH 7.4, containing 5% nonfat dried milk) and then probed with the primary antiphospho-ERK1/2, antitotal ERK1/2, anti-AGT, or mouse antibata actin antibody in a blocking buffer at 4°C overnight. The membranes were washed extensively in Tris-buffered saline containing 0.1% (v/v) Tween-20 before incubation for 1 h with a secondary antirabbit antibody conjugated to horseradish peroxidase. Membranes were then washed and developed using an ECL substrate. Beta-actin or total ERK 1/2 were used as a loading control.

### Statistical analysis

Statistical analysis was performed using one-way analysis of variance with Dunnett’s posttest. Each Fig represents one of at least 3 separate experiments. Results were expressed as mean ± standard error (SE). Data were analyzed using Student’s *t* test or analysis of variance, as appropriate. A value of *P* < 0.05 was considered significant for all tests.

## Results

### Serum starvation induced apoptosis of human umbilical vein ECs and VSMCs

**Fig 1:**
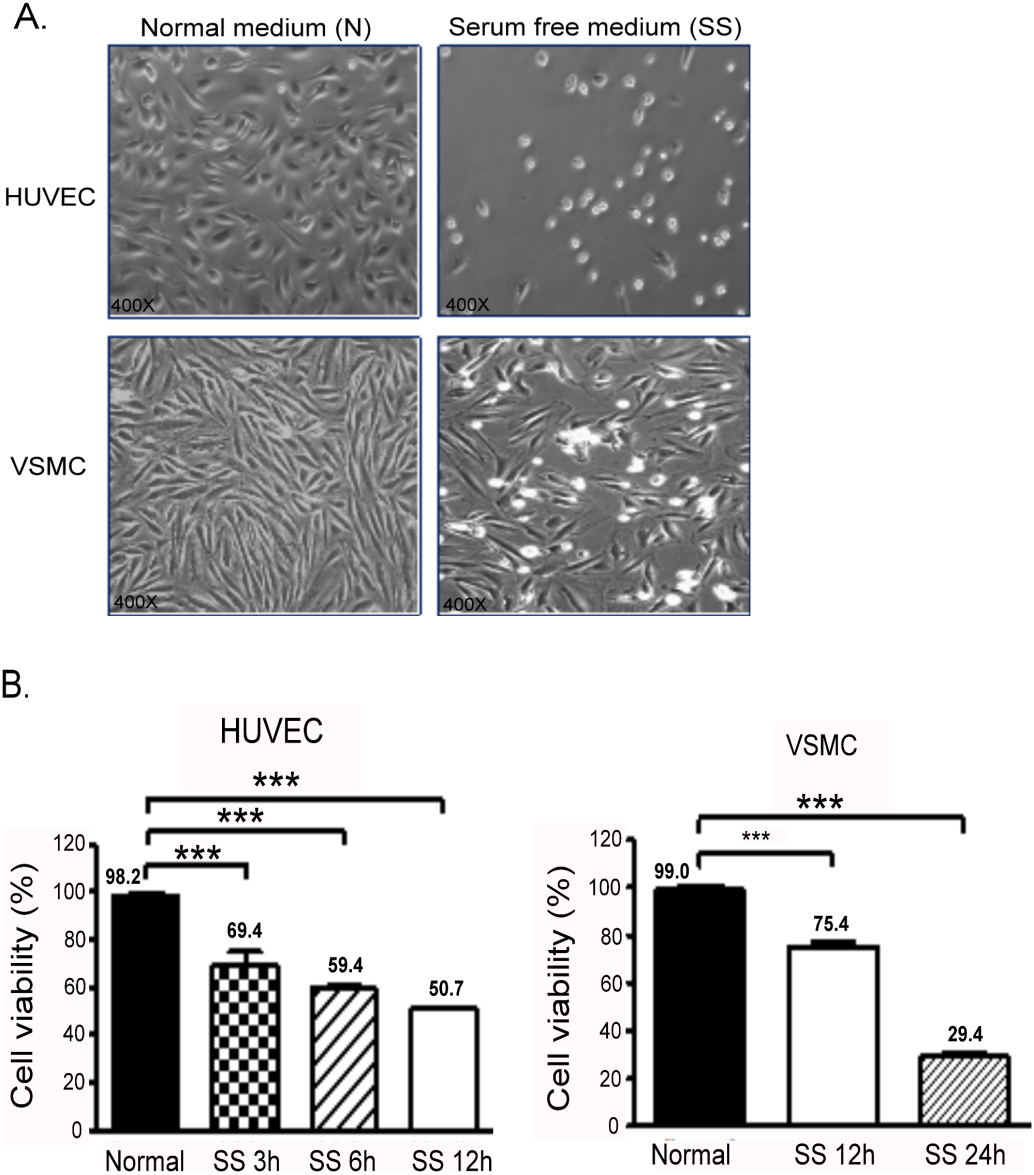
SS of HUVECs and VSMCs. (A) Effect of 24-h SS on cell morphology and density of HUVECs and VSMCs (right panel). (B) Cell viability was assessed after SS in HUVECs (at 3, 6, and 12 h) and in SMCs (at 6 and 12 h). The mean percentages of at least 3 independent experiments are represented in both cells. The square insert indicates the magnified area. ***P < 0.001 in comparison to cells without SS. Data are expressed as the mean ± SE and are representative of 3 experiments.

To imitate the condition that causes cell apoptosis in vitro, HUVECs and A7r5 VSMCs were starved using a serum-free medium. Both HUVECs and VSMCs underwent apoptosis or died after 24 h of SS (Fig 1A). Furthermore, the cell viability of HUVECs decreased rapidly to 65% after the cells underwent SS for 3 h. The cell viability of VSMCs also decreased after SS treatment (Fig 1B).

**Fig 2:**
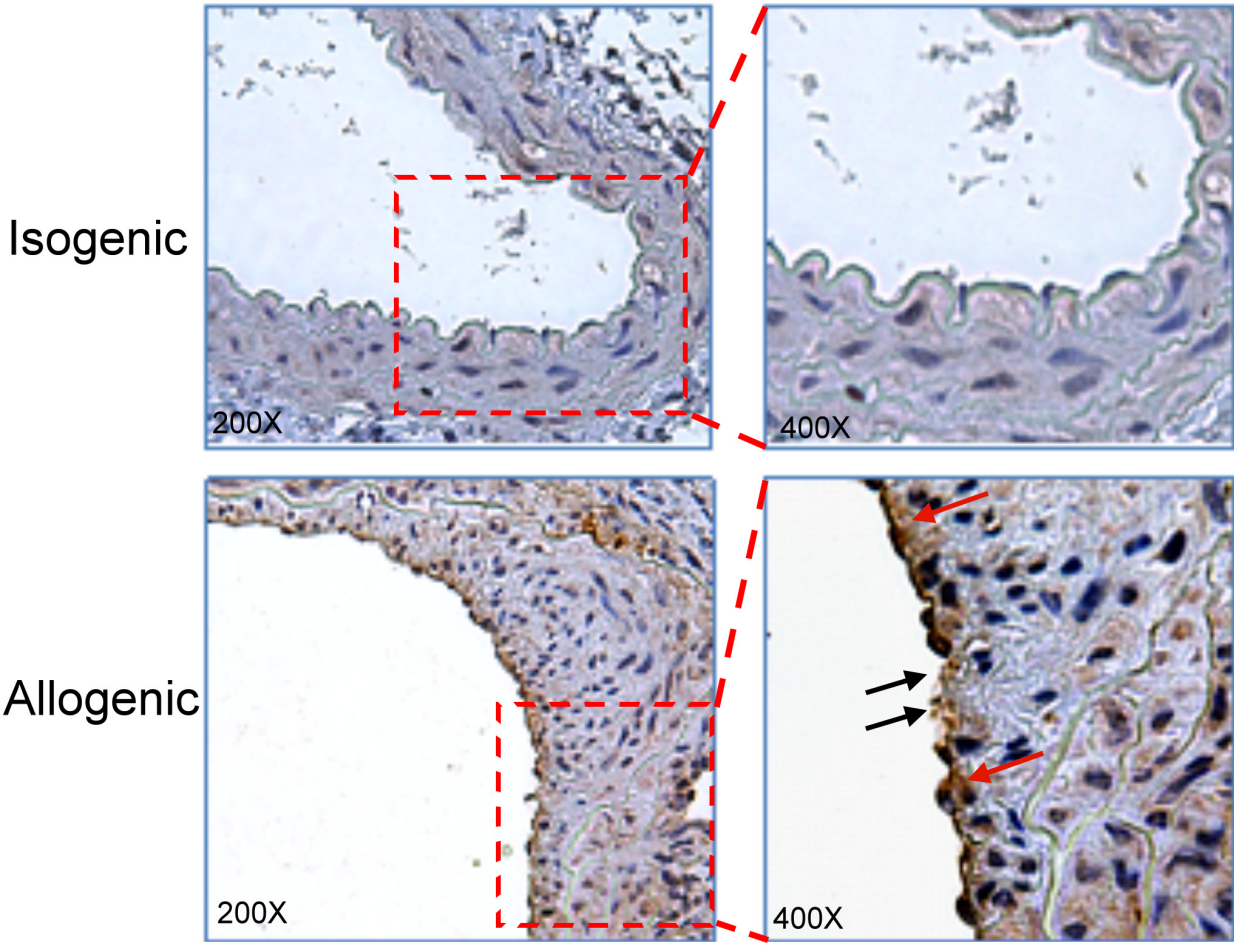
Apoptosis of ECs in an allograft vessel. Recipient and donor aortic sections stained for activated caspase-3 at 3 wk posttransplantation. Red arrows indicate activated caspase-3-positive cells; black arrows indicate fragmented and condensed DNA as well as apoptotic membrane blebbing of the endothelium. The square insert indicates the magnified area.

Neointima formation was found in a donor arterial segment of an allograft vessel, and fragmented and condensed nuclei and apoptotic EC blebbing were found in allogenic endothelia (Fig 2), in which the apoptotic ECs in the inner lining of the remodelling vessel were substantially stained with an activated caspase-3 antibody at 3 wk posttransplantation.

### AGT and ACE gene expression in apoptotic HUVECs and VSMCs

**Fig 3:**
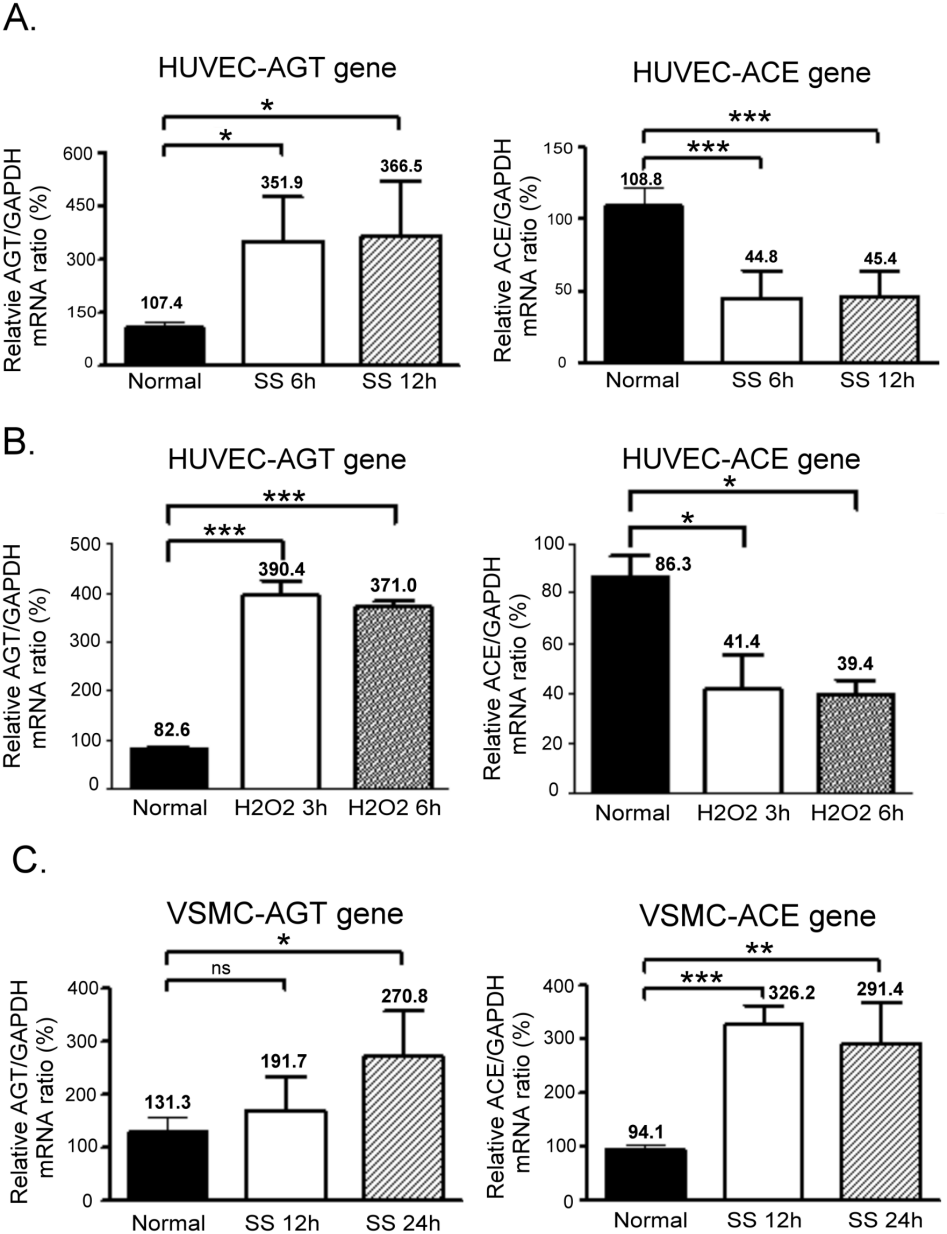
AGT and ACE gene expression in apoptotic HUVECs and VSMCs. (A) AGT and ACE gene expression in HUVECs that underwent SS for 6 and 12 h. (B) AGT and ACE gene expression in HUVECs exposed to H_2_O_2_ for 3 and 6 h. (C) AGT and ACE gene expression in VSMCs that underwent SS for 12 and 24 h. Real-time PCR was performed in the triplicate on each SS-treated sample for both AGT mRNA and ACE mRNA. The relative mRNA ratios represent the respective ΔCt of AGT or ACE normalised according to the ΔCt of GADPH in each experiment. The relative mRNA ratio in the normal medium was designated as 100%. Each assay was repeated 3 times, and the error bars in each individual figure represent the SE. *P < 0.05, **P < 0.01, and ***P < 0.001 in comparison to the control group. ns, no significance.

To examine the expression of AGT and ACE genes in response to SS in HUVECs, we extracted the total mRNA from the cells, which were incubated under SS conditions for 6 and 12 h, to perform a real-time qPCR assay. We found that SS significantly increased the expression of AGT mRNA by >3-fold (*P* < 0.05) in HUVECs compared with that in cells in a normal medium. By contrast, the expression of ACE mRNA decreased to approximately 50% (Fig 3A). A similar result was found when HUVECs were treated with 200 uM H_2_O_2_ (Fig 3B). We conducted the same experiment by using VSMCs and found that AGT mRNA was slightly increased in SS-treated VSMCs at the 12th hour and increased to 2.5-fold at the 24th hour compared with the control group. In contrast to the HUVECs, the expression of ACE mRNA in VSMCs incubated in the SS medium for 12 and 24 h was significantly upregulated to approximately 300% (Fig 3C).

### Apoptotic-stress-enhanced promoter activity of AGT and ACEs in VSMCs

**Fig 4:**
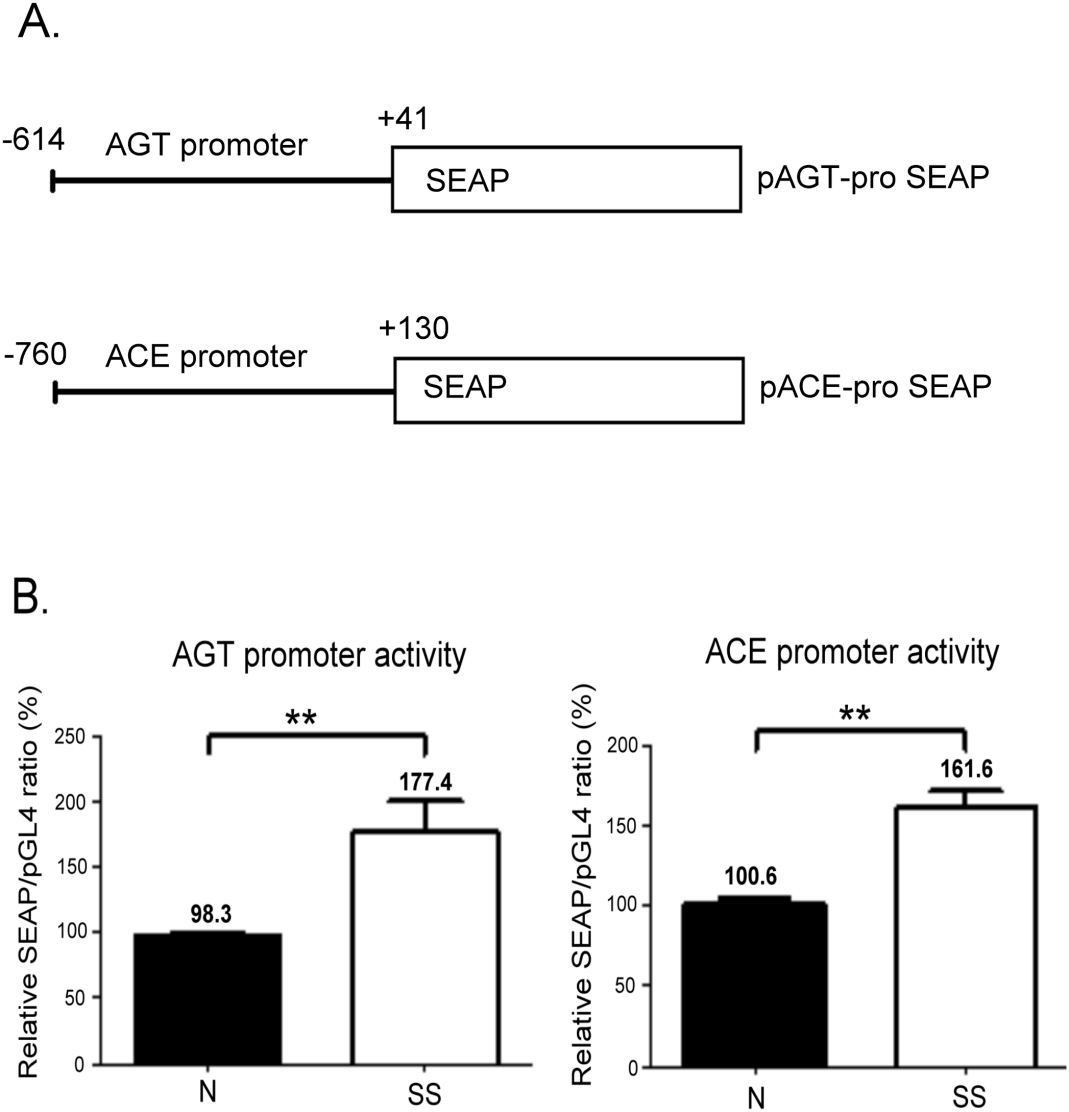
Promoter activity assay of human AGT and ACE genes in VSMCs. (A) Schematic diagrams of the reporter gene constructs. The AGT promoter (-614 to +41) and ACE promoter region (-760 to +130) were cloned into the upstream of the SEAP gene to generate the pAGT-proSEAP and pACE-proSEAP constructs, respectively. (B) SS treatment regulates the transcriptional activity of AGT and ACE promoters in VSMCs. pGL-4 was used as an internal control and contransfected with pAGT-proSEAP or pACE-proSEAP constructs in VSMCs. The relative SEAP/pGL-4 ratios represent SEAP activity normalised according to the firefly luciferase activity. The ratio in the normal medium was designated as 100%, and the values are expressed as the mean ± SE from at least 3 independent experiments. **P < 0.01 in comparison to cells cultured in a normal medium.

To confirm that the transcriptional activity of AGT and ACE genes is regulated by SS, we conducted a transient transfection in VSMCs by using reporter vectors cloned with the promoter region of an AGT and ACE, respectively (Fig 4A). After the cells were incubated in a normal or SS medium for 24 h, the transcriptional activity of both promoters was enhanced by SS in VSMCs (Fig 4B).

### Increased AGT and Ang II Expression was found in an SSC medium

**Fig 5:**
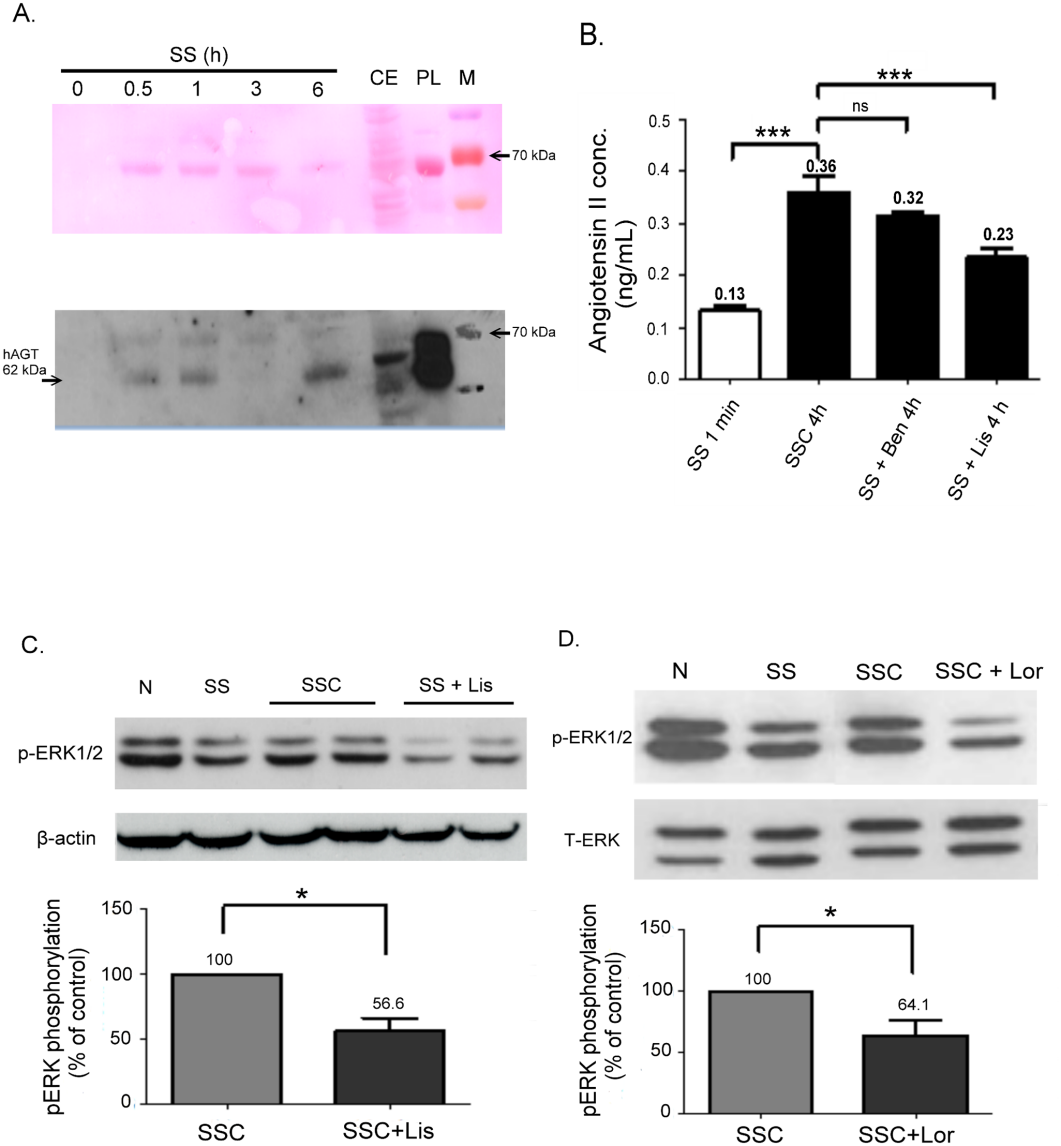
Detection of AGT and Ang II in a conditioned medium. (A) Western blot analyses of AGT expression in an SS medium with different incubation times (0 minutes, 30 minutes, 1 h, 6 h). CE, cell extract from HUVECs. PL, plasma from blood sample. M, ladder marker. Upper panel shows rapid (5 minutes) staining of protein bands on a PVDF membrane with the Ponceau S stain. (B) The Ang II concentration detected using an ELISA assay was highly increased in the SS medium (conditioned for 4 h) compared with that in the ECs conditioned in the serum-free medium for 1 minute (***P < 0.001). Conditioning ECs with lisinopril in an SS medium, SS+Lis, suppressed the production of Ang II (***P < 0.001), but suppression was not found in SS+Ben. ns, no significance. Data are expressed as the mean ± SE and are representative of 3 experiments. (C) Western blot analyses of phosphorylated ERK1/2 expressions in VSMCs exposed to N, SS, SSC, and ACEi-SSC (SS+Lis) for 1 h. The activation of phospho-ERK1/2 was suppressed in the ACEi-SSC group. (D) The activation of phospho-ERK1/2 in SSC was suppressed by 10 μM losartan.

Because of the high expression of AGT mRNA in starved HUVECs, we determined whether AGT protein was secreted into the SSC medium by using an immunoblotting assay. The AGT protein was detected in the serum-free medium when HUVECs were incubated with SS for 30 minutes; however the protein was not found with the original SS (at the 0-minute line) (Fig 5C). Rode plasma AGT is a heterogeneous protein that migrated at approximately 62 kDa on SDS-PAGE, as previously reported [18]. Moreover, we further assessed whether Ang II was produced in SSC by using a commercial ELISA kit. In a parallel experiment, another 2 ACEi-SSC media, SS+Lis and SS+Ben, were examined. We found that Ang II was significantly detected in SSC compared with SS (*P* < 0.001, Fig 5B), and the production of Ang II in SSC was significantly inhibited in SS+Lis (*P* < 0.001). However, the inhibition of Ang II was not observed in SS+Ben.

### Lisinopril inhibits Ang II production in an SSC Medium

On the basis of our previous findings that SSC activates ERK phosphorylation in VSMCs [14], we examined the activation of the ERK1/2 signal by treating the VSMCs with normal, SS, SSC, and SS+Lis media for 1 h. The SS+Lis medium blocked the phosphorylation of ERK1/2 activated by SSC (Fig 5C), indicating that lisinopril inhibited the Ang II produced in SSC. Similarly, we found that losartan inhibited the activation of ERK1/2 by SSC (Fig 5D).

## Discussion

RAS has been identified as being involved in the pathogenesis of neointima formation, and the upregulated expression of RAS components has been found in vascular lesions for more than a decade [6, 19–21]; however, the precise source of local augmented Ang II remains undetermined. We first demonstrated that the expression of AGT mRNA is highly enhanced in both ECs and VSMCs that were stressed by SS, and that the secretion of AGT protein and Ang II significantly increased in the SSC medium. Our findings were different from those of previous reports indicating that AGT increased only in the intima and neointima and that AGT mRNA was located predominantly in the periaortic brown adipose tissue and adventitia [18, 22]. Because EC apoptosis is a major initial event in neointima formation, the paracine molecules released from the programming of EC apoptosis are inferred to be vital regulators in the pathogenesis of neointima formation. The augmented AGT released directly from apoptotic ECs and nearby VSMCs could serve as another crucial precursor molecule of the paracrine effector, Ang II, which might synergetically function with LG3 to induce the migration and proliferation of VSMCs. The biological effects of Ang II are mediated mainly by the Ang II type 1 receptor (AT1R), and together they constitute one of the most crucial pathways in VSMC proliferation. In addition, it has been proven that the activation of ERK through AT1 signaling is associated with Ang II-mediated proliferation in VSMCs [23]. Furthermore, our previous result also showed that the ERK1/2 activated by SSC plays a crucial role in the migration and proliferation of VSMCs [14].

**Fig 6:**
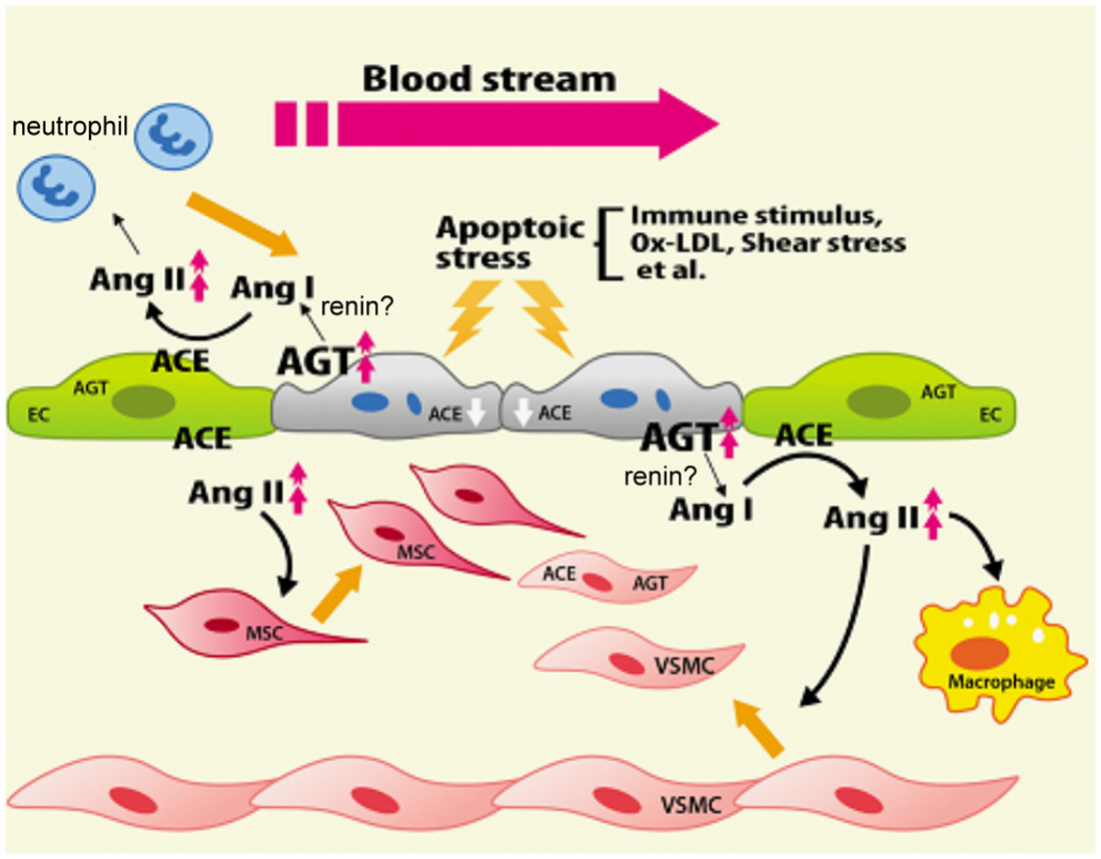
Model of initial crosstalk among ECs and surrounding cells in vessel lesions. The paracrine source of local augmented Ang II produced from apoptotic ECs serves as a vital messenger in the formation of neointima. ECs, endothelial cells. MSCs, mesenchymal stem cells.

Identifying the differences in AGT and ACE protein expression in the extremely thin lining of ECs in vivo is challenging, particularly when the cells undergo apoptosis. Thus, detecting the increased contents in the SSC medium was crucial. Because of the rate-limiting role of AGT in the RAS [24, 25], the release of an AGT protein as a progenitor of messengers from ECs might provide an efficient biological mechanism for informing neighbour cells to respond to apoptotic stress. ACE mRNA was suppressed in stress-induced apoptotic ECs. Although the exact mechanism through which the contrary expression of ACE and AGT genes is regulated remains unclear, it may be crucial for ECs to secrete priority mediators because ECs function on the surface of the vasculature and encounter continual and dynamic challenges from the blood stream in a vascular vessel. Nevertheless, ECs still require converting enzymes to produce Ang II peptides. Our study showed that ACE inhibitors inhibited the augmentation of Ang II in SSC, indicating that ACE or other converting enzymes are present in SSC or on the membranes of surviving HUVECs. We suggest that the amount of ACE should be sufficiently abundant in the surrounding ECs that survived to produce detectable Ang II in SSC because tissue-bound or secreted ACE has been shown to be produced by the endothelium of healthy human blood vessels [26, 27]. Moreover, consistent with previous reports indicating that increased AGT and ACE were found in the cellular structures of the neointima [21, 27, 28], we found that the mRNA expression and promoter activity of AGT and ACE genes were increased in apoptotic VSMCs. In addition, Ang II was shown to positively activate AGT expression by binding to AT1R in VSMCs [29]. Accordingly, Fig 6 shows a hypothesised model of local RAS crosstalk among ECs and their surrounding cells in vessels responding to apoptotic stress. However, the precise proportion of Ang II from the systemic or locally secreted part required to affect neointima formation requires further investigation because the synergistic contribution of AGT to its pathophysiological functions occurs in both systemic and cell-specific manners [30, 31].

The beneficial effect of an RAS blockade on cardiovascular events has been shown in several clinical trials and animal models [32–34]. Among RAS blockers, the administration of ACE inhibitors to reduce the acute sequelae of atherosclerosis and transplant rejection has been shown in some excel studies [35, 36]. Thus, the inhibition of ACE activity in both ECs and adventitial cells is an efficient potential strategy for treating the injured vasculature. The inhibition of ACE both suppresses the migration and proliferation of smooth muscle cells and reduces EC dysfunction [27]. The results of the present study consistently indicated that pretreatment with lisinopril could suppress the production of Ang II in the SSC medium, theoretically reducing the proliferation and migration of smooth muscle cells by prohibiting the activation of ERK1/2. However, this inhibition was not significantly found in the treatment of benazepril, which may be because of the difference of the binding affinity and pharmacodynamic efficacies between the 2 ACE inhibitors [37–39].

The limitation of the present study is that serum starvation culture condition would develop either cellular apoptosis or senescence. However, the factors that cause this differential outcome remain unclear. Following our previous reports and experimental designs [14], the apoptotic HUVECs increased substantially after the cells were treated with serum-free medium. Thus, we did not further differentiate between the cellular apoptosis and senescence. Besides, the transfection efficiency of the HUVEC delivery of the reporter vectors into the cells was extremely low. The reporter assays for AGT and ACE promoter activity were performed only in VSMCs. Examining the mechanisms of how these genes are oppositely regulated by transcription factors in ECs may provide further insight for researching the pharmacological control of AGT gene expression in apoptotic stress.

In conclusion, in addition to LG3, which is released through the degradation of an extracellular matrix of apoptotic ECs [14], the present study demonstrated that the augmented AGT released from apoptotic ECs and VSMCs plays a vital role as the progenitor of the messenger, Ang II, to accelerate the progress of vascular remodelling. Our findings fill the elusive gap between the initial event of EC apoptosis in the injured vasculature and the upregulated expression of partial local RAS components detected in the vessel lesion area. Consequently, selectively blocking the expansion of activated VSMCs is an advantageous strategy for reducing intimal hyperplasia. The clarification of initial paracrine factors involved in the development of vascular remodelling and the elucidation of apoptotic signaling cascades in lesion tissues can provide successful therapeutic strategies for preventing atheromatous vascular disease.
